# Synthesis and Electrospraying of Nanoscale MOF (Metal Organic Framework) for High-Performance CO_2_ Adsorption Membrane

**DOI:** 10.1186/s11671-016-1798-6

**Published:** 2017-01-05

**Authors:** Wahiduzzaman, Kelsey Allmond, John Stone, Spencer Harp, Khan Mujibur

**Affiliations:** 1 Department of Mechanical Engineering, Georgia Southern University, Statesboro, GA 30458 USA; 2 Department of Chemistry, Georgia Southern University, Statesboro, GA 30458 USA

**Keywords:** MOF, PAN, Electrospinning, Nanofibers, Electrospraying, CO_2_ adsorption

## Abstract

We report the sonochemical synthesis of MOF (metal organic framework) nanoparticles of 30–200 nm in size and electrospraying of those particles on electrospun nanofibers to process a MOF-attached nanofibrous membrane. This membrane displayed significant selectivity towards CO_2_ and capacity of adsorbing with 4000–5000 ppm difference from a mixed gas flow of 1% CO_2_ and 99% N_2_. Applying ultrasonic waves during the MOF synthesis offered rapid dispersion and formation of crystalline MOF nanoparticles in room temperature. The MOF nanoparticles of 100–200 nm in size displayed higher surface area and adsorption capacity comparing to that of 30–60 nm in size. Nanofibrous membrane was produced by electrospinning of MOF blended PAN solution followed by electrospraying of additional MOF nanoparticles. This yielded uniform MOF deposition on nanofibers, occurred due to electrostatic attraction between highly charged nanoparticles and conductive nanofibers. A test bench for real-time CO_2_ adsorption at room temperature was built with non-dispersive Infrared (NDIR) CO_2_ sensors. Comparative tests were performed on the membrane to investigate its enhanced adsorption capacity. Three layers of the as-produced membranes displayed CO_2_ adsorption for approximately 2 h. Thermogravimetric analysis (TGA) of the membrane showed the thermal stability of the MOF and PAN up to 290 and 425 °C, respectively.

## Background

The continuing demand for energy around the world is a primary reason for utilizing available resources such as fossil fuels, coals etc. at extremely high rates. As a result, large amounts of hazardous gases are released into the environment which has become a major global concern for the environmentalists [1]. Carbon dioxide, a leading proponent of Greenhouse effect is the matter of concern especially due to its rapid increase in the atmosphere. The recently reported CO_2_ concentration in the atmosphere has been found as 404 ppm with an alarming increasing rate of 2.9 ppm/year [2].

In the world of nanotechnology, a significant amount of research has been conducted over the last few years to produce an effective methodology to adsorb CO_2_ gas. Mishra and Ramaprabhu suggested a system of magnetite multi-walled carbon nanotubes which were prepared by a catalytic chemical vapor deposition method followed by purification and functionalization. The functional results proved that this composite material system worked fine in absorbing CO_2_ gas under high pressure and temperature [3]. Activated carbons (ACs) and zeolite-based molecular sieves have shown good performance in high CO_2_ adsorption capacities [4]. Electric swing adsorption system also drew further attention. In this process, a cycle of seven steps (feed, rinse with hot CO_2_-rich stream, internal rinse, electrification, depressurization, and purge) ensured CO_2_ absorption procedure from flues gases of natural gas power station [5]. Using Grand Canonical Monte Carlo simulations for modeling, effective absorption of hydrogen-methane mixtures in idealized single-walled nanotubes had been observed. Performance analysis of these kinds of nanotubes was done in different pressure, along with room temperature [6]. In case of post-combustion gas capturing, properties, and qualities of nanomaterials gave a viewpoint on interesting and highly effective absorption capacity for CO_2_ [7]. Nanomaterials, therefore, are considered to be highly potential in CO_2_ capturing due to their large surface areas and adjustable properties. Recently, solvent stripping by ammonia is known as an effective way to absorb high amount of CO_2_ [8]. Several other different kinds of nanomaterials including nanocrystalline NaY zeolite, ZnO, MgO nanoparticles as well as mixed phase aluminum nanowhiskers have been investigated for adsorption analysis [9]. Nanocrystalline zeolites possess high external surface area and active sites present on the external surface to adsorb a significant amount of CO_2_ gas [10]. The nano-adsorption materials both have pros and cons, as discussed by Wang et al. [11]. CaO nanopods comparatively have higher selectivity towards CO_2_, eventually displayed higher adsorption capacity. Carbon nanotubes (CNT) functionalized with nanofibers have higher surface area but less selective to CO_2_.

Metal organic frameworks (MOF) are crystalline porous materials constructed by metal ions and organic ligands. Considered to be a breakthrough material for gas adsorption purpose, MOFs possess three dimensional crystalline structures formed by the coordination bond between metal based salts and organic ligands. Properly synthesized and tuned MOF particles exhibit high surface area and porosity, making them able to act as a gas storage tank. It offers unchanged and optimized gas uptake capacity. Molecular level tuning and functionalization of the MOF particles are required to improve the adsorption capacity and selectivity towards certain gas [12]. They possess crystallographically well-defined robust 3D structures with extremely large surface areas compared to volume. CO_2_ binding on adsorption sites can also be further enhanced by incorporation of unsaturated metal centers, metal doping, and chemical functionalization. Other tunable properties such as low energy regeneration, stability in the presence of moisture, and various operating conditions have shown much promise in the utilization of MOFs as physisorbent or chemisorbent materials on an industrial scale [13]. When the MOF crystals started to form inside the precursor solution, small micro- and nano-pores have been formed on the crystal surface. When the solvents are evaporated, these pores have become open and acted as pathways of gas capturing access [14]. Synthesis of MOF has usually been done by solvothermal process which consists of mixing the specific metal salt and organic linker for a certain period of time and post-processing afterwards to attain the desired microcrystalline porous structures [15]. Sonochemical method has also been reported for MOF synthesis. The method of generating ultrasonic waves through the precursors offers a rapid and homogeneous nucleation of the particles, forming MOF [16]. This method is proven to be effective to achieve reduced particle size with well-defined crystallography [17]. SEM, TGA, XRD, and Raman Spectroscopy are some of the well-known morphological and characteristic analysis to investigate the crystallinity and CO_2_ adsorption performance of MOF [18]. As high temperature synthesis of MOF had found to produce undesirable by-products such as metal oxides, room temperature synthesis has become a considerable solution for that [19]. Surface and size control of MOFs have also drawn much attention for research endeavors. The initially synthesized MOF crystals size was mostly confined to big micron size particles. Using ultrasonic waves, Tehrani et al. produced nanorod-like shaped HKUST-1 crystals [20]. Using microwave radiation has also proved to be useful to produce nano MOFs. Comparative analysis of Ni and Mg-based MOFs between typical solvothermal and microwave approach displayed exceptional reduction in MOF size and shape [21]. Klinowski et.al also adopted microwave synthesis but instead of radiation, they opted for microwave heating which also allowed short reaction times, fast kinetics of crystal nucleation and growth, and high yields of desirable products which can be isolated with few or no secondary products [22]. Several other unique approach have been undertaken to synthesize nano size MOF crystals. Sanchez et al. had come up with spray-drying methodology to dissemble a HKUST-1 MOF encrust into nano-MOF crystals [23]. This spray-drying strategy enables the construction of multi-component MOF superstructures and the encapsulation of guest species within these superstructures.

Electrospinning is a versatile and most widely used and preferred process to produce sub-micron and nanoscale polymeric fibers. A polymer solution of sufficient viscosity and moderately high molecular weight is drawn from a spinneret under the influence of a high voltage electric field. The influence of electrostatic force and surface tension on the solution droplet helps it stretch into continuously formed nanoscale fibers. During the spinning, the solvent solution gets evaporated, and solid electrospun fibers are collected in a collector placed underneath [24]. Free-standing MOF membrane can be produced on electrospun fibrous mats for gas adsorption purpose. Different types of MOF crystals such as HKUST-1, ZIF-8, and MIL-101 have been used to fabricate the membrane [25]. However, most MOF particles were seen encapsulated in the fibers, thus made it inefficient for CO_2_ adsorption. Composite novel kind of nanofibers with a loading of maximum 40% MOF were reported via electrospinning, it was also observed that the conjugation of the MOF and polymer-derived fibers became difficult due to the increasing MOF percentage into the materials [26]. Highly porous nanofibers have been prepared by electrospinning MOF (metal–organic framework) nanoparticles with suitable carrier polymers. Nitrogen adsorption proved the MOF nanoparticles to be fully accessible inside the polymeric fibers [27]. Functionalizing polymer surfaces with MOF particles have been found difficult because of the unavailability of finding a way of attaching MOF particles on the fiber surface. Centrone et al. performed in situ microwave irradiation to grow MOF particles into the polymer surfaces [28]. The particles were seen mostly agglomerated or discretely dispersed on the fiber surface, making the substrate almost invisible. With the formation of small MOF nanoparticles attached to the electrospun fiber substrates, it is possible to increase the gas uptake capacity of the membrane. Using coordination modulation method, size of MOF particles was reduced to nanoscale [29]. Atomic layer deposition (ALD) and anionic treatment of precursor fibers have made it possible to attach large amount of nano MOF particles on the substrate fibers [30, 31]. However, ALD process is costly and anionic treatment is not suitable for strong polymer-based fibers such as PAN. Therefore, further emphasis should be given on generating an applicable and cost-effective method of fabricating nanofibrous CO_2_ adsorption membrane. Our previous approach to produce a MOF-loaded adsorption membrane consists of electrospun PAN (polyacrylonitrile) membrane loaded with MOF particles of 3–6 μm in size [32]. HKUST-1 was selected as MOF because of its excellent adsorption performance and compatibility with PAN. This is a Cu-based MOF (empirical formula C_18_H_6_Cu_3_O_12_), typically known to have octahedral crystalline structure. The formation of the HKUST-1 is highly influenced by the precursors, solvents, synthesis method, and post-processing. Cu(NO_3_)_2_ was chosen as the primary precursor because of its stronger characteristic peaks found in the XRD patterns over CuCl_2_ and Cu(CH_3_COOH)_2_ [33]. In this work, a new approach of conjugating electrosprayed HKUST-1 nanoparticles on electrospun nanofibers is reported in order to produce a nanofibrous membrane for enhanced CO_2_ adsorption performance.

## Experimental Methods

### Synthesis of MOF Nanoparticles

HKUST-1 was selected as the MOF to be conjugated with the electrospun nanofibers to produce the adsorption membrane. Sonochemical approach of MOF synthesis was carried out by mixing 2.55 gm of Cu(NO_3_)_2_.3H_2_O salt and 0.45 gm of Trimesic acid (1,3,5-benzenetricarboxylic acid) in a 200-mL solvent mixture of DMF, ethanol, and DI water (1:1:1) with an addition of 1 mL of TEA (Triethylamine) as deprotonating agent. The mixture was sonicated in room temperature for different time period of 30, 60, and 120 min. The sonication yielded blue HKUST-1 crystals during the synthesis which were later extracted and washed with the mother liquor three times via centrifuging. The obtained MOF crystals were then dried in a vacuum oven at 120 °C for 18 h to ensure complete evaporation of the remaining solvents and activation.

### Electrospinning of MOF Hybridized Nanofibers

**Fig. 1:**
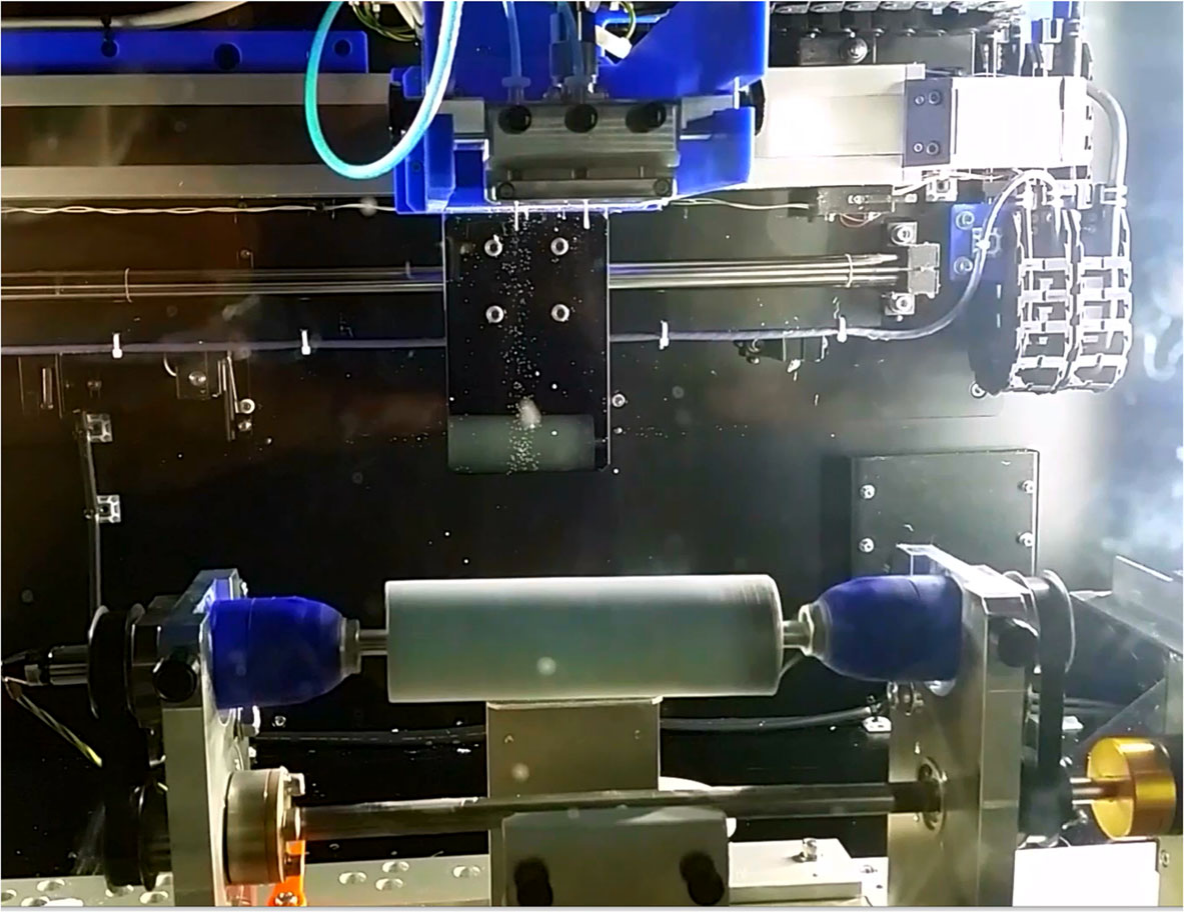
Electrospinning of HKUST-1 hybridized PAN nanofibers

In a 33 mL solution of DMF, 0.45gm (15 wt% of PAN) of HKUST-1 was added and started mixing using a sheer mixer at 70 °C. An amount of 3 gm of PAN was added to this solution slowly. The mixing was carried out for 3 h, eventually made a blue precursor solution of PAN with MOF embedded inside. Two syringes of 6 mL each of the prepared solution were placed into the NF-500 electrospinning unit. Multi-jet spinneret was used during the electrospinning process. The flow rate applied was 1.2–1.4 ml/h and voltage was 24 to 26 KV. Using multi-jet spinneret, it made possible to run the spinning of the two solutions at the same time. This produced a continuous streamline of nanofibers drawn from both of the needles which were collected on a cylindrical porous canister model as shown in Fig. 1. The collector was kept rotating at a speed of 120–140 rpm, and the distance between the syringe needles and the collector was kept in between 150 and 170 mm. Relative humidity was kept at 30–40% because of PAN’s sensitivity to water or moisture. This eventually produced MOF hybridized non-woven PAN nanofibrous membrane, wrapped around the canister model. The color of the membrane is bluish-white, contrary to plain white color of neat PAN fiber mat. The fiber canister was then dried in a vacuum oven at 50 °C for 3 h.

### Electrospraying of HKUST-1 Nanoparticles on Nanofibrous Membrane

Electrospraying is a method of liquid atomization by means of electrical forces. The liquid during electrospraying flows out of a capillary nozzle, maintained at an extremely high voltage. The particle formation is forced by the electric field to be dispersed into fine droplets. In this work, 1 wt% of the previously synthesized HKUST-1 nanoparticles was dispersed in ethanol by sonicating for 15 min, forming a stable MOF suspension. Using multi-jet spinneret, the solution was then electrosprayed at an extremely high voltage of 40–45 KV. In order to achieve that high voltage in the electrospinning unit, the collector-needle distance had to be maintained at 150 mm or above. A high flow rate of 3.5–4 ml/h was implied during the electrospraying process. The process was carried on for 5 h. This approach was proved to be effective for MOF conjugation, eventually led to a uniform blue colored membrane. The membrane was kept in vacuum oven and dried at 45 °C for 2 h to influence rapid crystallization of the MOF particles.

### CO_2_ Adsorption Test

**Fig. 2:**
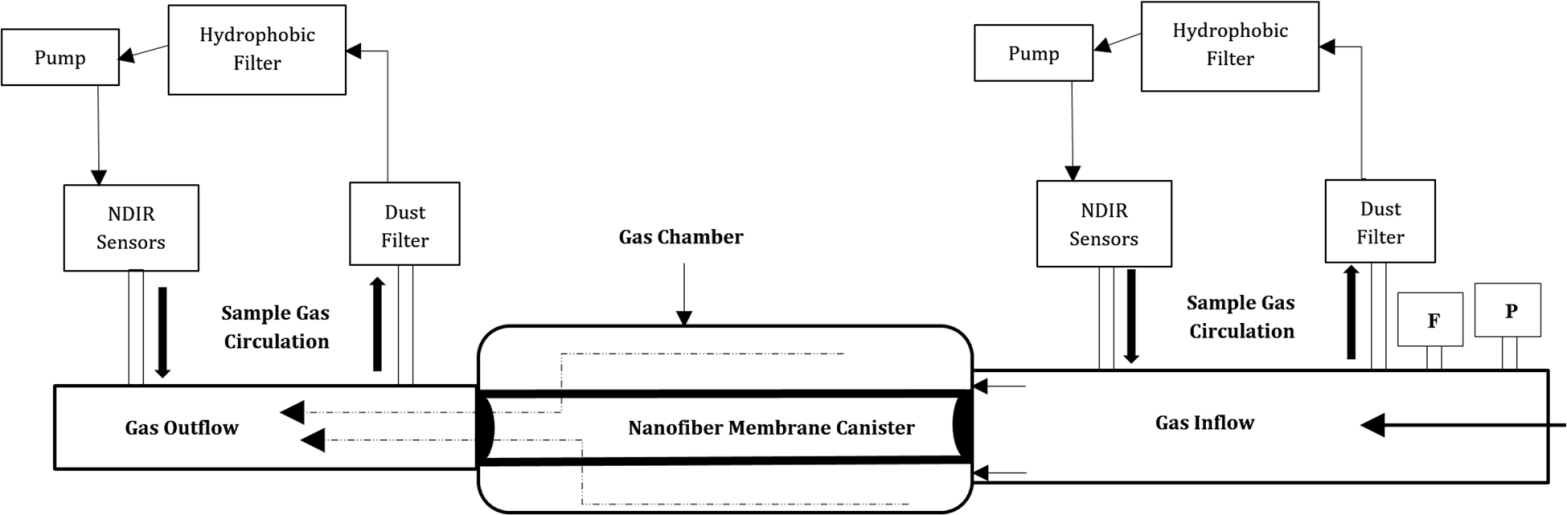
Schematic diagram of CO2 adsorption test setup

A test setup was built for real-time CO_2_ adsorption at room temperature. The schematic diagram of the setup is showed in Fig. 2. The setup consists of a PVC-made cylindrical gas chamber. The membrane canister was placed into the chamber and tightly sealed with 3D printed sealing caps. Two NDIR (non-dispersive infrared) CO_2_ sensors, purchased from CO_2_ meter, were placed at the inlet and outlet ports. The sensors came with a dust filter and a hydrophobic filter. Sample gas was drawn into the sensors by a motor driven pump. The sensors read the concentration of CO_2_ in the sample gas in ppm (parts per million). Real-time data of CO_2_ concentration can be plotted from the sensors by using GASLAB software. The accuracy of the sensors is roughly ±70 ppm [34]. They were calibrated at zero point with a calibration gas of known gas concentration. It takes 50 s to stabilize and get fully diffused by the calibration gas. A gas tank contains 1% CO_2_ and 99% N_2_ was used for gas inflow.

The adsorption tests were performed in room temperature. The total volume of the test chamber was calculated as 1278.2 cm^3^. Methodology of the test consists of fill the gas chamber for a certain period of time, letting the filter membrane to adsorb CO_2_ and refill it again after releasing the previous gas inflow. Any disturbance or vibration, or movement of the test setup was not required because of causing fluctuation in the CO_2_ reading. Elementary adsorption can be detected by the real-time plot difference between the CO_2_ values at inlet and outlet.

## Results and Discussion

### HKUST-1 Nanoparticles

**Fig. 3:**
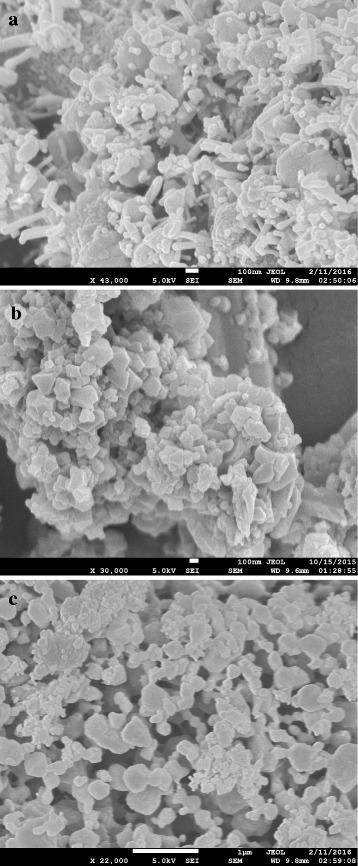
SEM images of the HKUST-1 particles synthesized by (**a**) 120, (**b**) 60, and (**c**) 30 min of sonication

The sonochemical synthesis of HKUST-1 produced significant size reduction in MOF particles thus achieving higher surface area and increased gas adsorption performance. The ultrasonic waves during sonication caused fast dispersion and disintegration of precursor materials, which led to a homogenous reaction and formation of smaller MOF particles at room temperature within a short period of time. Using TEA as nucleation agent during synthesis influenced the rapid deprotonation of the organic linker, resulting in homogenous nucleation and reduction of the particle size [35]. The obtained crystals were found to be in nanoscale after washing and post-treatment. A 2 h of sonication eventually produced HKUST-1 crystals of 30–60 nm (Fig. 3a). But an hour of sonication produced fine octahedral crystals of 100–200 nm (Fig. 3b). Figure 3c showed nanocrystals of 400–600 nm synthesized from a sonication period of only 30 min. Table 1 shows the effect of sonication time period on the nanocrystals size distribution.

**Table 1:** Experimental data of HKUST-1 synthesis or different sonication time

Sonication time (hour)	Addition of TEA (mL)	Post-heating time (hour)	Yield (%)	Particle size (nm)
2	1	12	79	30–60
1	1	15	64	100–200
0.5	1	18	62	400–600

**Fig. 4:**
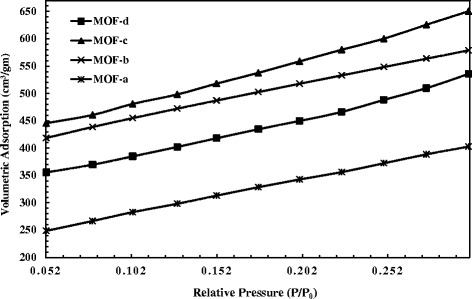
Adsorption isotherms of the HKUST-1 samples

**Fig. 5:**
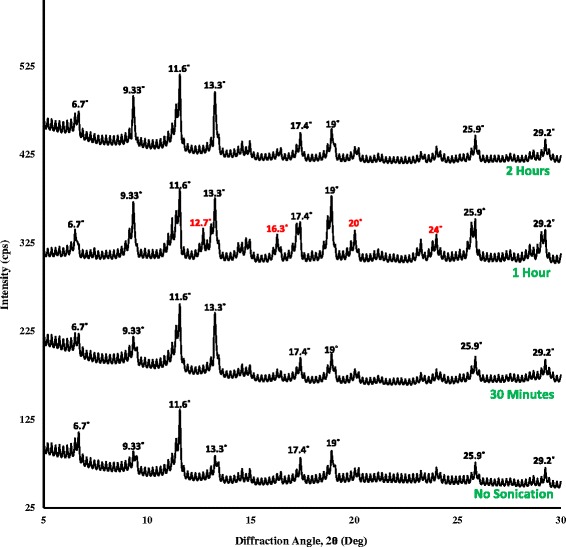
XRD patterns of HKUST-1 samples at different condition for sonication

On the one hand, conventional solvothermal method is known to yield large micron-size MOF crystals. Solvothermal synthesis of HKUST-1 particles (2–6 μm) had also been reported in our previous work [32], which has taken into account in here as well for a comparative BET analysis between that and the newly synthesized particles by sonication. Table 2 provides the data of surface area and maximum volumetric N_2_ adsorption of the HKUST-1 samples, and Fig. 4 shows the adsorption isotherms of those samples. The isotherm for HKUST-1 sample of an hour of controlled sonication (MOF-c) showed the largest increasing pattern followed by MOF-b and MOF-d. The smallest pattern was observed for MOF-a, synthesized by solvothermal method. From Table 2, it was also clearly seen that the sonochemical samples displayed higher surface areas with notable increase in N_2_ uptake capacity comparing to that of solvothermal method. The highest surface area (2025 m^2^/gm) was achieved by MOF-c which also displayed typical octahedral crystalline structure of HKUST-1. On the other hand, solvothermal approach displayed HKUST-1 particles of significantly lower surface area of 1095 m^2^/gm which occurred due to the prolonged heating of the precursors at high temperature. The crystallization of MOF in solvothermal method carried on as long as the particles remained in the solution, thus continued their surface augmentation. In addition, there were unwanted by-products such as Cu_2_O which remained in the pores of the structure. It was also observed from the BET tests that, although increasing sonication time produced smaller particles, this also subsequently reduced the surface area and the volumetric capacity of the MOF nanoparticles when sonication time increased from 1 to 2 h. The XRD diffraction patterns of different HKUST-1 samples with different sonication time are shown in Fig. 5. Characteristic peaks of HKUST-1 at 6.7°, 9.33°, 11.6°, 13.3°, 17.4°, and 19° were found, which appeared to be similar with the work reported by Wang et.al and Biemmia et.al [36, 37]. Samples prepared by using sonication displayed sharp characteristic peaks compared to the samples with no sonication. It is also observed that, the intensity of the peak at 9.33° has increased with increased sonication time. More importantly, it has been found that the XRD pattern of HKUST-1, sonicated for an hour, showed the well-defined characteristic peaks at 12.7°, 16.3°, 20°, and 24°, which is not present with the other categories of samples. We believe the samples with 1 h sonication have presence of more crystalline mixed phases when compared to the other categories of samples.

**Table 2:** Particle size and surface area of the HKUST-1 samples

Sample	Method	Particle size	Surface area (m^2^/gm)	Volumetric N_2_ adsorption (cm^3^/gm)
a	Solvothermal	2–6 μm	1095	403
b	Sonochemical (2 h)	30–60 nm	1797	579
c	Sonochemical (1 h)	100–200 nm	2025	651
d	Sonochemical (0.5 h)	400–600 nm	1539	535

### Electrospinning of HKUST-1 Blended PAN Nanofibers

**Fig. 6:**
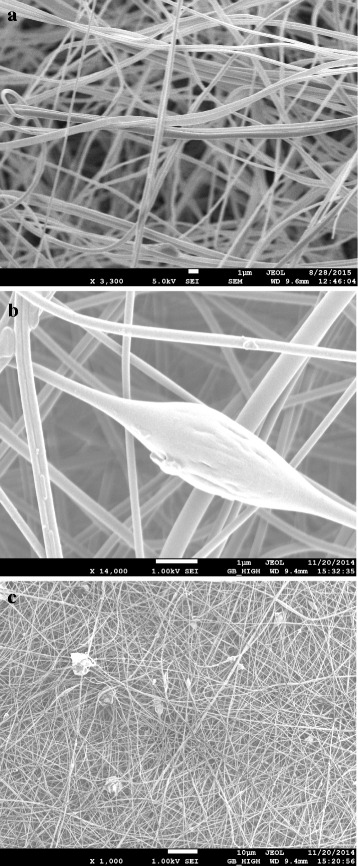
SEM images of (**a**) neat PAN nanofibers, (**b**) HKUST-1 impregnated nanofibers, and (**c**) discrete HKUST-1 particles around the fiber membrane

Electrospinning of PAN nanofibers displayed non-woven nanofibrous white outlook (Fig. 1). When HKUST-1 particles were included in the PAN precursor solution, the nanofibers appeared to be bluish-white. The inclusion can be found either as impregnated inside the fiber or non-uniformly distributed along the fiber surface. From SEM image of neat PAN nanofibers in Fig. 6a, well-smoothed fibers can be seen without any beads or particles seen anywhere. On the other hand, Fig. 6b shows impregnation of MOF material found inside the spun fiber. Figure 6c shows a general view of the MOF-loaded nanofibers, showing presence of particle distributed along the fiber surface. The presence of MOF particles can even be increased in the PAN fibers, but higher loading of MOF eventually affected the electrospinning process. Instead of having continuous fibers, undesirable flakes and droplets were found because of the presence of larger MOF crystals. The impregnated and dispersed MOF particles in the fibers are for creating seed layers and contact points for additional MOF inclusion. Nevertheless, the as-produced fiber mat would not be proven effective for gas adsorption purpose because of low amount of MOF particles and lower total surface area. Therefore, an additional approach of electropsraying HKUST-1 nanoparticles was undertaken.

### Electrospraying of HKUST-1 Nanoparticles on the PAN Membrane

**Fig. 7:**
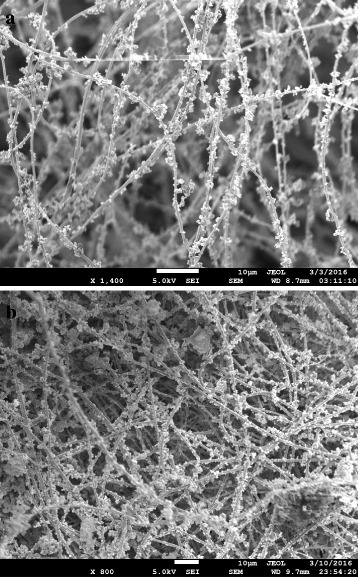
SEM images of (**a**) nano-MOF electrosprayed functional nanofibers and (**b**) enhanced nano-MOF attachment on the nanofibers

The rapid sonochemical synthesis of nanoscale HKUST-1 paved the way of formulating new methodology conjugating MOFs with nanofibrous membrane. The nano HKUST-1 particles showed a comparatively more stable suspension in ethanol. Electrospraying of the MOF particles at the extremely high voltage with faster flow rate made the particles plausible to be accumulated on the previously electrospun nanofibers with strong attachment. The MOF particles during electrospraying became highly charged, eventually deposited on the conductive PAN fibers with strong attachment. This attachment was due to the strong electrostatic attraction between the charged MOF particles and conductive PAN nanofibers. The higher flow rate of electrospraying also influenced the MOF deposition. Figure 7a showed the SEM image of the MOF-loaded nanofibers where nano HKUST-1 particles are seen distinctively attached to individual fibers, appeared as a necklace-like structure. The typical crystal structure of HKUST-1 was also evident in those conjugated HKUST-1 nanoparticles. Figure 7b showed a substantial amount of MOF conjugated with the fibers, achieved by a three-hour duration of continuous electrospraying.

### BET Analysis of the Adsorption Membrane

**Fig. 8:**
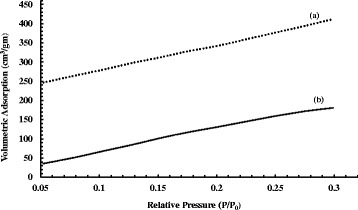
Nitrogen adsorption isotherm by (**a**) nano-MOF electrosprayed fiber membrane displaying a maximum uptake of 412 cm3/gm and (**b**) solvothermally synthesized MOF-loaded fiber membrane displaying a maximum gas uptake capacity of 180 cm3/gm at maximum pressure

The sonochemically synthesized nano-MOF electrosprayed fiber membrane showed an increasing surface area of 1217 m^2^/gm and a pore volume of 0.53–0.56 cm^3^/gm. The maximum N_2_ adsorption capacity of the membrane was 412.23 cm^3^/gm. The values are significantly larger than the fiber membrane reportedly produced using the MOF particles synthesized by solvothermal method [32]. A comparative BET analysis between the two differently produced membranes is given in Fig. 8.

### Real-time CO_2_ Adsorption Test

**Fig. 9:**
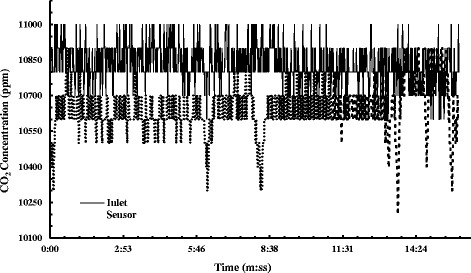
Adsorption test for neat PAN nanofiber membrane

**Fig. 10:**
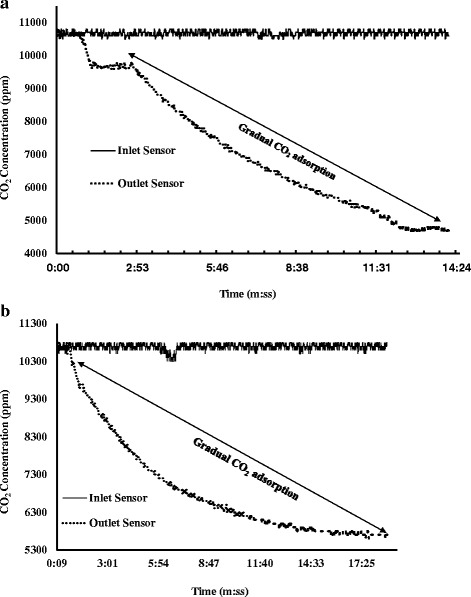
Experimental plot of breakthrough CO2 adsorption in MOF electrosprayed fiber membrane for (**a**) 10 and (**b**) 20 min

The purpose of the breakthrough CO_2_ adsorption experiment was to determine the real-time gas adsorption performance of the fabricated membrane. Leakage tests have been performed to ensure no leaks in the enclosed setup. The flow rate of the gas inflow to the cylindrical chamber was 94 cm^3^/s for 13 s, and the working pressure was kept at 48.26 kPa. Each cycle of breakthrough testing contained entrapment of mixed gas in the gas chamber and allowing the fiber membrane placed inside to adsorb it. First test was run on neat PAN nanofiber mat for over 10 min. It was observed that no adsorption took place inside the test chamber as both the inlet and outlet sensors gave the data relates to 1% CO_2_ (Fig. 9). In Fig. 10a, the first run of adsorption test for the electropsrayed fiber membrane is shown. The gradual degradation at the outlet sensor reading started displaying after 35 s of initialization. The test was run for almost 10 min with a total difference of 6100 ppm between the inlet and outlet sensor reading. The percentage of reduction of CO_2_ was 35.38%. Test 2 was carried on for the electrosprayed membrane for a longer time to observe the gas uptake capacity of the product. A maximum gas loading at the chamber at 118 cm^3^/s rate was undertaken for 30 s. The pressure was increased to 55.15 kPa. The declining pattern at the outlet sensor was noticed after 25 s of initialization. After 22 min, the gas concentration showed a total difference of 5200 ppm, showed in Fig. 10b. The percentage of reduction of CO_2_ was 28.65%. The total adsorption time for the new membrane after a few more similar experiments was found to be almost 80 min, before the membrane led into saturation with filled gas. CO_2_ molecules are known to have a larger quadrupolar moment and smaller kinetic diameter comparing to N_2_. This results in strong interaction between CO_2_ and the open metal sites of MOF with higher binding energy [38]. Moreover, the crystal structure of HKUST-1 has a substantial selectivity factor of 7:1 towards CO_2_ over N_2_ in room temperature at around 35 kPa [39]. This also corroborates the CO_2_ selectivity and superior adsorption performance of HKUST-1.

**Fig. 11:**
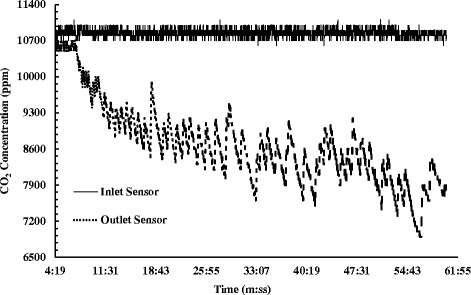
CO2 adsorption data for open channel gas flow

An open channel test was performed as well for the electrosprayed fiber membrane. For longer time of adsorption, a layer-by-layer fabrication of the MOF-loaded fiber membrane was undertaken. There were, overall, three layers of nanofibrous membrane were produced on a single canister model. The thick membrane was then used for the adsorption test in an open channel, operating at a low pressure of 1–2 psi and a reduced flow rate of 5–6 ft^3^/h. As found from Fig. 11, the open channel test displayed a different pattern of CO_2_ reading in the outlet sensor. The ppm reading in the outlet followed a decreasing trend with several peaks. This pattern was found due to the continuous flow of gas which allowed the fiber membrane less time and contact sites to capture and store CO_2_. The adsorption took place for a significantly longer period of almost 102 min, gradually lowering the ppm level to 6500 ppm before the membrane became saturated. This testing approach signifies the increasing adsorption efficacy and prospective usefulness of the fiber membrane at different open gas outlet sources.

### Thermogravimetic Analysis (TGA) of the Membrane

**Fig. 12:**
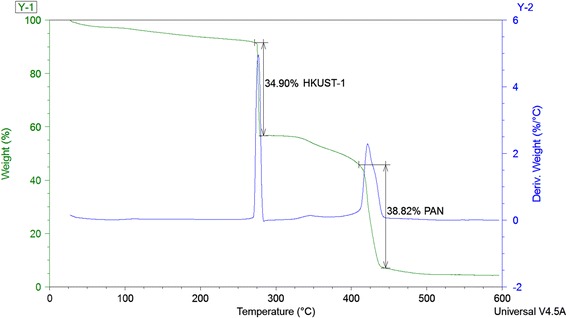
TGA analysis showing 35% weight loss for the HKUST-1 at 270–290 °C and 39% loss of PAN at 400–430 °C

Thermogravimetric analysis (TGA) was performed on the HKUST-1/PAN membrane to determine the thermal stability and elemental analysis. The TGA was carried on up to 650 °C at a heat rate of 5 °C/min. The fiber membrane is assumed to be functional in high temperature and rough environment. From Fig. 12, it was observed that the sample had a gradual degradation of element degradation. Two sharp peaks of 35 and 39 wt% loss of material were observed in the temperature range of 270–290 °C and 400–430 °C, respectively. The first peak signified the weight loss of HKUST-1 nanoparticles which was found to be similar with the work of Wang et al. [40]. The second peak verified the thermal stability of electrospun PAN nanofibers for a higher temperature range, similar to what was reported by Chauque et.al [41]. It was also observed that, the thermal decomposition of the previously reported micron-scale MOFs occurred from 150–220°, which signified the increased thermal stability for the nanoscale MOF particles. The overall thermal stability of the nano-MOF-loaded membrane therefore, can be functional at harsh environment.

## Conclusion

In this work, HKUST-1 MOFs were synthesized at room temperature by sonochemical method where, instead of typical solvothermal heating, the precursor solution was sonicated for a certain period of time. The resultant product was blue HKUST-1 nanoscale crystals of 30–200 nm. The nanocrystals were washed multiple times and heated at 100–120 °C for 18 h to ensure activation of the product for adsorption. The smaller MOFs offered substantially larger BET surface area, up to 2025 m^2^/gm. Electrospinning of HKUST-1 blended PAN precursor produced a nanofibrous membrane which was used as substrates for additional MOF loading. The nanofibers were contained with MOF particles, showing small degree of impregnation and dispersion in the nanofibers. The membrane was used as substrate for additional MOF inclusion. By applying electrospraying of the nano-MOF particles on the fiber substrates, a novel functional fiber membrane was produced. SEM images of the fiber samples showed distinctive attachment of MOF nanoparticles with the fiber body, appeared to be necklace-like structure. By electrospraying for a longer time, the amount of MOF attached to the fiber body was significantly increased. The BET analysis of this membrane displayed a surface area of 1217 m^2^/gm and maximum N_2_ adsorption of 412 cm^3^/gm. The nanofibrous membranes were then placed into a test bench for testing the adsorption capacity. A mixed gas tank containing 1% CO_2_ was used for the experiment. Two infrared CO_2_ sensors were connected at the inlet and outlet of the test bench to determine the difference of CO_2_ concentration in ppm. Test 1 showed a difference of 6100 ppm within a timeframe of 10 min, and test 2 showed a difference of 5200 ppm after being ran for 22 min. The electrosprayed membrane showed notably enhanced CO_2_ capturing performance, operated for a longer time period of total 80 min. Three layers of electrosprayed MOF fiber membrane were then carried out. The membrane was undertaken for an open gas flow channel at a very low pressure. The adsorption in the membrane took place for almost 102 min before getting saturated. The outlet sensor plot displayed a slowly decreasing trend with several peaks. X-ray diffraction analysis of the MOF nanoparticles showed characteristic HKUST-1 pattern with specific peaks. From the TGA analysis, it was certain that the fiber membranes have good thermal stability with a decomposing temperature at 270 °C.

## Abbreviations

BET: Brunauer–Emmett–Teller
MOF: Metal organic framework
PAN: Polyacrylonitrile
SEM: Scanning electron microscopy
TGA: Thermogravimetric analysis
XRD: X-ray diffraction
